# Retinal Ganglion Cell Loss and Microglial Activation in a SOD1G93A Mouse Model of Amyotrophic Lateral Sclerosis

**DOI:** 10.3390/ijms22041663

**Published:** 2021-02-07

**Authors:** Pilar Rojas, Ana I. Ramírez, Manuel Cadena, José A. Fernández-Albarral, Elena Salobrar-García, Inés López-Cuenca, Irene Santos-García, Eva de Lago, José L. Urcelay-Segura, José M. Ramírez, Rosa de Hoz, Juan J. Salazar

**Affiliations:** 1 Instituto de Investigaciones Oftalmológicas Ramón Castroviejo, Universidad Complutense de Madrid, 28040 Madrid, Spain; pilar.rojas.lozano@gmail.com (P.R.); airamirez@med.ucm.es (A.I.R.); joseaf08@ucm.es (J.A.F.-A.); elenasalobrar@med.ucm.es (E.S.-G.); inelopez@ucm.es (I.L.-C.); ramirezs@med.ucm.es (J.M.R.); 2 Instituto Oftálmico de Madrid, Hospital General Universitario Gregorio Marañón, 28007 Madrid, Spain; cadenamd@gmail.com (M.C.); joseluis.urcelay@salud.madrid.org (J.L.U.-S.); 3 OFTARED-ISCIII, IIORC, Universidad Complutense de Madrid, 28011 Madrid, Spain; 4 Departamento de Inmunología, Oftalmología y ORL, Facultad de Óptica y Optometría, Universidad Complutense de Madrid, 28037 Madrid, Spain; 5 Departamento de Bioquímica y Biología Molecular, Facultad de Medicina, Instituto Universitario de Investigación en Neuroquímica, Universidad Complutense de Madrid, 28011 Madrid, Spain; isantosg@ucm.es (I.S.-G.); elagofem@med.ucm.es (E.d.L.); 6 Centro de Investigación Biomédica en Red de Enfermedades Neurodegenerativas (CIBERNED), Instituto Ramón y Cajal de Investigación Sanitaria (IRYCIS), 28040 Madrid, Spain; 7 Departamento de Inmunología, Oftalmología y ORL, Facultad de Medicina, Universidad Complutense de Madrid, 28040 Madrid, Spain

**Keywords:** microglia, retina, SOD1G93A mouse model, ALS, retinal whole-mount, microglial activation, retinal ganglion cells, pro-inflammatory M1 phenotype, anti-inflammatory M2 phenotype

## Abstract

The neurodegenerative disease amyotrophic lateral sclerosis (ALS) affects the spinal cord, brain stem, and cerebral cortex. In this pathology, both neurons and glial cells are affected. However, few studies have analyzed retinal microglia in ALS models. In this study, we quantified the signs of microglial activation and the number of retinal ganglion cells (RGCs) in an SOD1G93A transgenic mouse model at 120 days (advanced stage of the disease) in retinal whole-mounts. For SOD1G93A animals (compared to the wild-type), we found, in microglial cells, (i) a significant increase in the area occupied by each microglial cell in the total area of the retina; (ii) a significant increase in the arbor area in the outer plexiform layer (OPL) inferior sector; (iii) the presence of cells with retracted processes; (iv) areas of cell groupings in some sectors; (v) no significant increase in the number of microglial cells; (vi) the expression of IFN-γ and IL-1β; and (vii) the non-expression of IL-10 and arginase-I. For the RGCs, we found a decrease in their number. In conclusion, in the SOD1G93A model (at 120 days), retinal microglial activation occurred, taking a pro-inflammatory phenotype M1, which affected the OPL and inner retinal layers and could be related to RGC loss.

## 1. Introduction

Amyotrophic lateral sclerosis (ALS) is a neurodegenerative disease that causes the loss of both lower and upper motor neurons [1,2,3,4,5]. Twenty percent of familial ALS cases are caused by SOD1 mutations, and the mode of inheritance is autosomal dominant [6,7]. In transgenic mice, when the mutant human Cu/Zn SOD1 enzyme is expressed at high levels, motor neuron disease occurs, indicating that the altered protein is the cause of the disease [8]. There are two possible pathogenic mechanisms involved in ALS SOD1 models. In the first, mutant SOD1 tends to aggregate into cytoplasmic inclusion bodies, altering motor neuron functions. In the second, the excess reactive oxygen species caused by mutant SOD1 cause oxidation of the proteins or lipids of motor neurons. In addition, oxidative damage of proteins can also induce protein aggregation [6]. Numerous strains of transgenic SOD1 mice have been created that differ in the types of their gene mutations and the levels of the expression of the mutant protein, which leads to variations in the expression of the disease [8,9,10]. In the SOD1G93A (tg (SOD1-G93A) Gur1) transgenic mice used in this study, the pathological changes are highly stereotyped. The animals begin to show symptoms at 3 months, and 4–5 months of age, complete paralysis occurs along with death [6,11]. The histopathological changes in these mice are characterized by vacuolizations in motor neurons [12]. At the end of the disease (4–5 months), there is a significant loss of lumbar and cervical spinal cord motor neurons, denervation of skeletal muscle, and loss of diaphragm inputs [11]. In these animals, there is also microglial activation at 100 and 120 days of age (3.5–4 months) in the cervical and lumbar spinal cord regions, which are reflected in the transcriptional alterations of genes involved in inflammation [6].

Neuroinflammation is a common pathogenic mechanism in many neurodegenerative diseases, such as Alzheimer’s disease, Parkinson’s disease, and glaucoma, among others [13]. Neuroinflammation involves the activation of microglial cells, which are the immune cells of the central nervous system and have the ability to respond to damage. When this activation occurs, the microglia can undergo morphological changes, as well as migrate and proliferate. In addition, activated microglia can exist broadly in two different activation phenotypes: M1 and M2. M1 produces an intense inflammatory response characterized by the release of pro-inflammatory cytokines (IFN-γ, TNF-α, IL-1β, IL-6, and IL-12) and inflammatory mediators (nitric oxide and reactive oxygen species). Uncontrolled activation of the M1 phenotype can lead to a state of chronic inflammation, which can induce neuronal death. However, the M2 phenotype produces the release of anti-inflammatory cytokines (IL-4, IL-10, IL-13, and TGF-β) and neurotrophic factors (BDNF, neurotrophins, GDNF, etc.), contributing to the control of inflammation and neuronal survival [13,14]. One of the specific markers of M2 is the enzyme arginase 1 (Arg1) [15].

Microglial activation also occurs in ALS, as seen in the mutant mice for SOD1, and on spinal cord samples from ALS patients, which can influence motor neuron damage [16,17,18]. It has also been demonstrated in primary motor neuron and glia cultures that exogenous mSOD1(G93A) does not cause directly detectable motor neuron death. However, it produces both morphological and functional activation of the microglial cells, which increases the release of pro-inflammatory cytokines and free radicals. In addition, when the microglia were co-cultured with motor neurons, extracellular mSOD1(G93A) was shown to cause motor neuron damage. These data suggest that extracellular mSOD1(G93A) is not directly toxic to motor neurons but requires microglial activation for its toxicity [19].

In recent years, it has become evident that ALS not only affects motor neurons, the spinal cord, the cerebellum, and the cerebral cortex, but also leads to alterations in the visual system, including the retina. Using optical coherence tomography (OCT), Roth et al. 2013 did not observe any involvement of the anterior visual pathway in ALS [20]. However, further studies have found alterations in retinal thickness in ALS patients, suggesting that these changes observed with OCT could be useful as biomarkers. Thus, decreases in retinal thickness have been observed, suggesting a process of neurodegeneration in the retina [21,22,23,24,25]. However, an increase in macular thickness in the temporal and lower areas of the inner macular ring has also been found in patients with early stages of the disease, suggesting that this phenomenon could be due to microglial activation during a neuroinflammatory process [26].

Studies that analyzed microglial cells in retinas with ALS are very scarce. To our knowledge, there have been only two studies conducted using two different models of ALS, and both presented contradictory results. Ringer et al. [27], in the SOD1G93A mouse model, found no microglial activation. However, Cho et al. [28] found an increase in microglial cells with a marked increase in amoeboid forms in a mouse model of ALS devoid of ran-binding protein2 (Ranbp2).

Therefore, in the present work, comparing the retinas of SOD1G93A mice at a very advanced stage of the disease (120 days) with age-matched control animals, we performed the following: (i) a quantitative and morphometric study of the microglial signs of activation (the area occupied by each microglial cell, process retraction, and cell number); (ii) an analysis of the phenotypes of microglial cells M1 (by labeling the microglial cells with anti- IFN-γ and anti-IL-1β) and M2 (by labeling them with anti-arginase-I and anti-IL-10); and (iii) a study of the number of retinal ganglion cells (RGCs) to determine if a neurodegenerative process is taking place. For this purpose, we used retinal whole-mounts, which allowed us to visualize the entire cell and its location along the X, Y, and Z axes in all retinal layers containing the cells being analyzed (microglia and RGCs).

## 2. Results

### 2.1. Qualitative Study of Iba-1+ Retinal Microglial Cells

**Figure 1:**
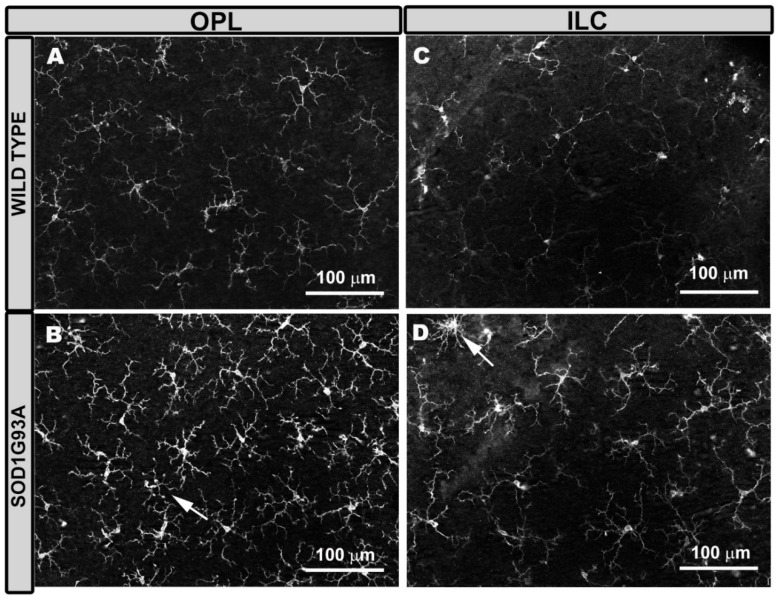
Microglial cells in the outer plexiform layer (OPL) and in the inner layer complex (ILC) constituted by the inner plexiform layer and nerve fiber layer–ganglion cell layer. Retinal whole-mount was labeled with anti-Iba-1. In aged-matched wild type mice, the microglial cells of the OPL (**A**) and the ILC (**C**) showed a ramified morphology, featuring primary processes from which they derive secondary processes, and constituted a regular plexus of tiled cells along the retina. ILC microglia processes were thinner than those of OPL. In SOD1G93A mice, the microglia of the OPL (**B**) and the ILC (**D**) showed thickening of the cell body and processes, giving the cell a more robust and larger appearance. Some microglial cells showed a retraction of these processes (arrow). Number of retinas used in the experiment, WT: *n* = 6; and SOD1G93A: *n* = 6.

The microglial cells of aged-matched wild-type (WT) mice were distributed along the entire retina in a regular mosaic fashion, separated from each other more or less equidistantly. These cells formed plexuses in the OPL (Figure 1A) and in the inner layer complex (ILC) (constituted by the inner plexiform layer (IPL) and the nerve fiber layer (NFL)–ganglion cell layer (GCL)) (Figure 1C).

However, in the photoreceptor outer segment layer (OS), microglial cells were very scarce and did not form a plexus, with only 0–2 cells found per retina. These cells had an ovoid soma from which numerous processes emerged from a single point. In the OPL and ILC, microglial cells had a triangular soma from which processes emerged. The processes were divided into primary (from 3 to 4), secondary, and tertiary and became finer as they were subdivided (Figure 1A,C).

**Figure 2:**
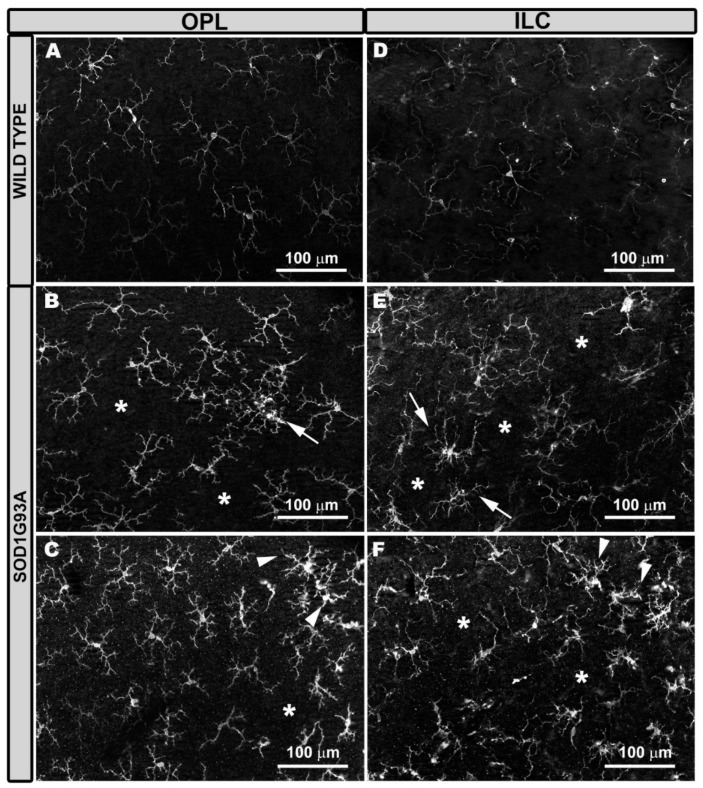
Microglial cells in the outer plexiform layer (OPL) and inner layer complex (ILC) constituted by the inner plexiform layer and nerve fiber layer–ganglion cell layer. Retinal whole-mount labeled with anti-Iba-1. Compared to wild type mice (**A,D**), in SOD1G93A mice the microglial plexus was not as regular in OPL (**B**,**C**) and in ILC (**E**,**F**). There were areas where the microglia grouped together and featured retracted processes, leaving areas without cells (*). The groups of cells were formed either in circles (**B**,**E**) (arrows) or in rows (**C**,**F**) (arrowheads). Number of retinas used in the experiment, WT: *n* = 6; and SOD1G93A: *n* = 6.

In SOD1G93A mice, the microglia were generally thicker (both the somas and primary and the secondary processes) (Figure 1B,D) than in the WT mice (Figure 1A,C), except in the OS layer. In the SOD1G93A group, the microglial tertiary processes were more difficult to distinguish, as they were observed as thickening of the secondary process itself. In these animals, the overall appearance of the cell was more robust and larger (Figure 1B,D). However, on some occasions, we observed cells with more retracted processes (Figure 1B,D). In the SOD1G93A group (Figure 2B,C,E,F), the microglial plexus was less regular than that in the WT group (Figure 2A,D). In the transgenic animals, we found, in some areas of the retina, clusters of microglial cells that formed circular areas (Figure 2B,E) or rows (Figure 2C,F), leaving the adjacent areas free of microglia (Figure 2B,C,E,F). In the cluster zones, the microglia had their processes retracted.

### 2.2. Expression of Microglial Phenotypes M1 or M2

**Figure 3:**
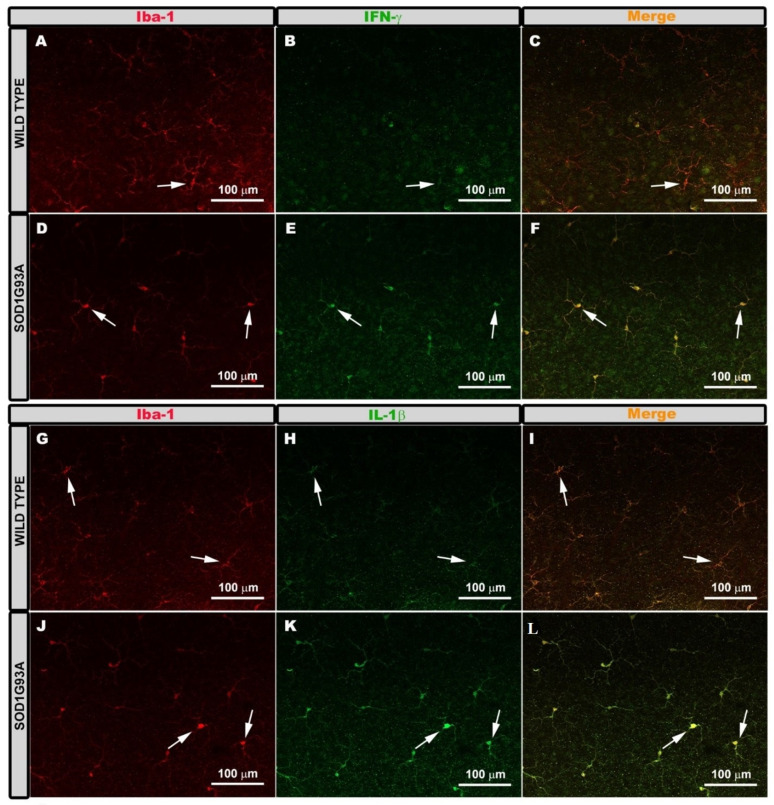
Pro-inflammatory M1 phenotype. Retinal whole-mounts are labeled with anti Iba-1 and anti IFN-γ (**A**–**F**) and with anti-iba-1 and anti-IL-1β (**G**–**L**) showing the microglial plexus in the outer plexiform layer. Immunoreactivity for IFN-γ (**A**–**C**) and IL-1β (**G**–**I**) in the Iba-1+ cells was very low in the wild type group (arrow). Iba-1+ cells of the SOD1G93A group showed very intense immunoreactivity for IFN-γ (**D**–**F**) and IL-1β (**J**–**L**) (arrows). Number of retinas used in the experiment, WT: *n* = 3; and SOD1G93A: *n* = 3.

To determine if Iba-1+ microglial cells showed characteristic markers of the M1 pro-inflammatory phenotype, we performed double immunostaining against Iba-1 and IFN-γ or IL-1β. In the WT group, Iba-1+ cells showed very low immunoreactivity for both antibodies, IFN-γ (Figure 3A–C), and IL-1β (Figure 3G–I). However, in the SOD1G93A group, Iba-1+ cells showed intense immunoreactivity for both antibodies, IFN-γ (Figure 3D–F) and IL-1β (Figure 3J–L), indicating an increase in their expression. This was confirmed by measuring the mean intensity value for both antibodies. In the WT group, the mean intensity values were for IFN-γ expression (12.23 ± 3.32) and for IL-1β (14.28 ± 3.73). In the SOD1G93A group, the mean intensity values were for IFN-γ expression (27.64 ± 7.45) and for IL-1β (31.02 ± 7.74).

**Figure 4:**
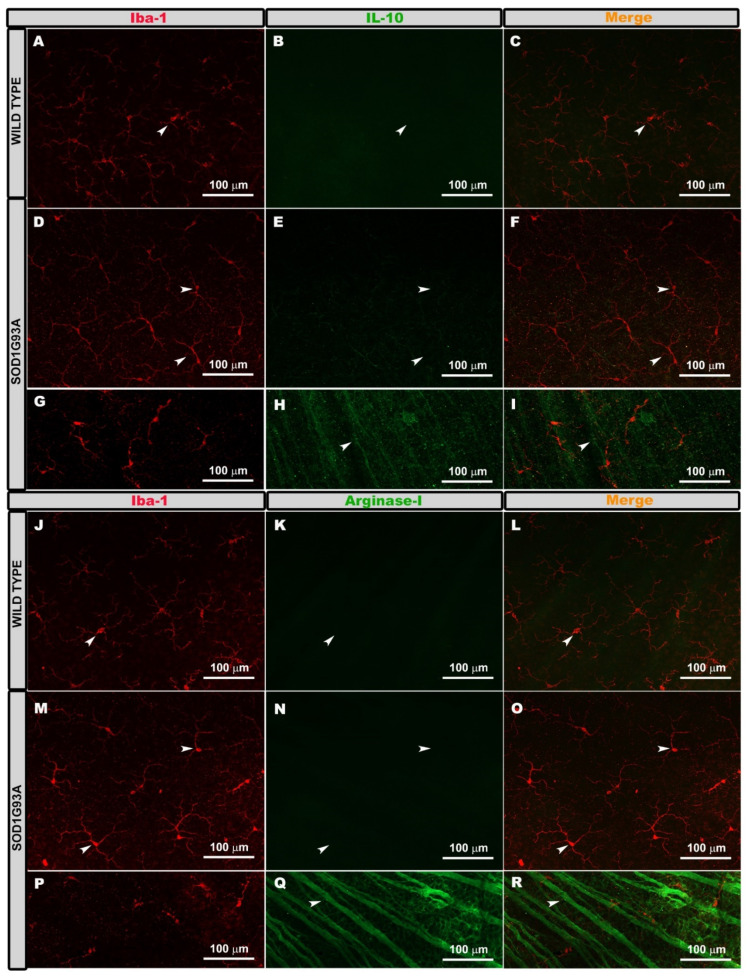
Anti-inflammatory M2 phenotype. Retinal whole-mounts labeled with anti Iba-1 and IL-10 (**A**–**F**) and with anti-iba-1 and arginase-I (**J**–**O**). Positive control for immunolabeling of IL-10 (**G**–**I**) and for arginase-I (**P**–**R**). The images (**A**–**O**) show the microglial plexus of the outer plexiform layer. The Iba-1+ cells were not labeled with IL-10 in either the wild type (**A**–**C**) or the SOD1G93A group (**D**–**F**) (arrows). They were also not labeled with arginase-I in the wild type group (**J**–**L**) or in the SOD1G93A group (**M**–**O**) (arrows). The axons of the retinal ganglion cells were labeled with IL-10 (**H**,**I**) (arrows) and with arginase-I (**Q**,**R**) (arrows). Number of retinas used in the experiment, WT: *n* = 3; and SOD1G93A: *n* = 3.

To analyze if the microglial cells Iba-1+ were immunolabeled with antibodies characteristic of the phenotype M2 (anti-inflammatory), we performed double immunostaining against Iba-1 and arginase-I or IL-10. In both the WT and SOD1G93A groups, the Iba-1+ cells were not marked with either of the two antibodies, IL-10 (Figure 4A–F) or arginase- I (Figure 4J–O). However, the RGC axons were labeled with both antibodies (IL-10 (Figure 4H,I) and arginase-I (Figure 4Q,R)). This indicated that the microglia did not express arginase-I or IL-10.

### 2.3. Quantitative Study of Iba-1+ Microglial Cells

#### 2.3.1. Number of Cells

**Figure 5:**
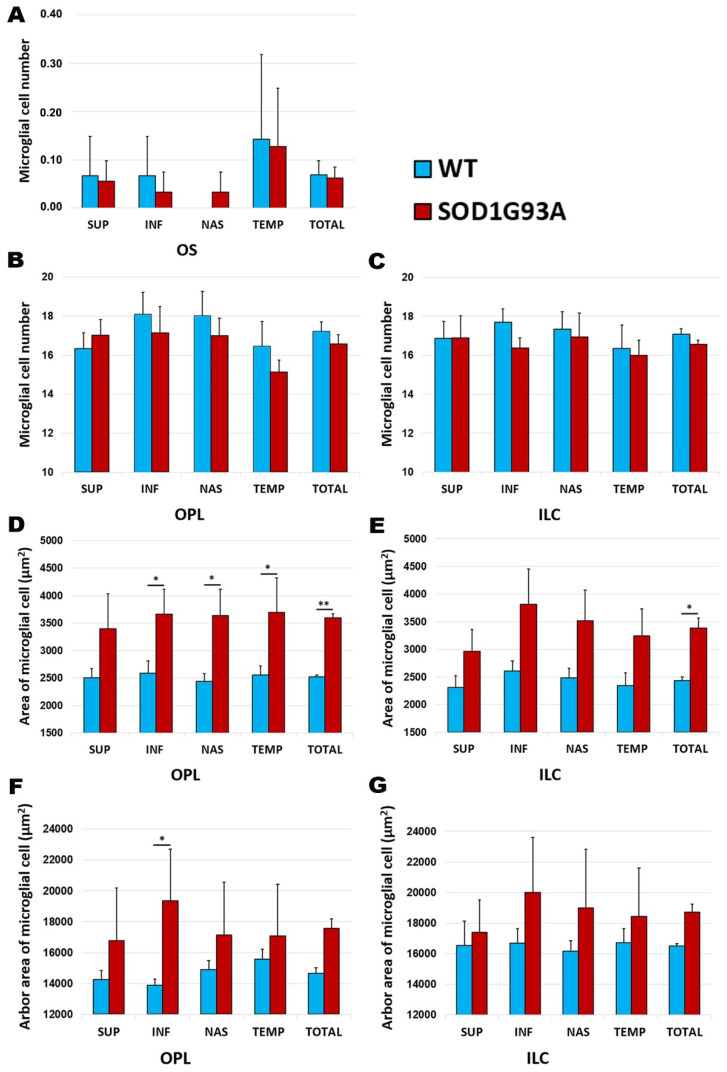
Morphometric analysis of the signs of microglial activation in the wild type group and the SOD1G93A group. (**A**–**C**) The number of Iba-1+ cells per area of 0.1502 mm^2^ in the OS (**A**), OPL (**B**), and ILC (**C**); (**D**,**E**) Quantitative analysis of the area of the microglial cells (µm^2^) in OPL (**D**) and ILC (**E**). (**F**–**G**) Quantitative analysis of the arbor area of the microglial cells (µm^2^) in OPL (**F**) and ILC (**G**). The histograms show the mean number (± standard deviation, SD). * *p* < 0.05, ** *p* < 0.01. Photoreceptor outer segment layer (OS), outer plexiform layer (OPL), and inner layer complex (ILC) (constituted by an inner plexiform layer and a nerve fiber–ganglion cell layer). Wild type (WT). Superior (SUP), inferior (INF), nasal (NAS), and temporal (TEM). Number of retinas used in the experiment, WT: *n* = 6; and SOD1G93A: *n* = 6. Statistical test: Mann–Whitney U test.

The comparison between the SOD1G93A and WT groups showed no significant differences in the number of microglial cells in any of the retinal layers: OS (Figure 5A), OPL (Figure 5B), or ILC (Figure 5C).

#### 2.3.2. Area of Microglial Cells

**Figure 6:**
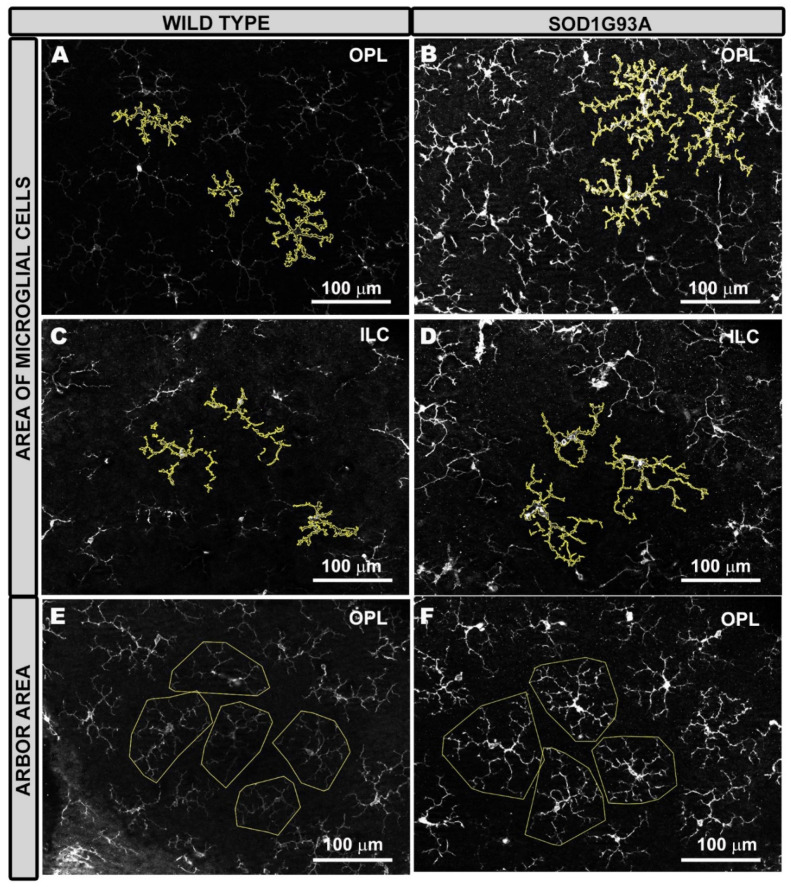
Representative images of the area of the microglial cells (**A**–D) and the arbor area (**E**,**F**) in the wild type (**A**,**C**,**E**) and SOD1G93A (**B**,**D**,**F**) groups. The area of microglial cells was significantly larger in the SOD1G93A group (**B**,**D**) than in the wild type (**A**,**C**) in both OPL (**A**,**B**) and ILC (**C**,**D**), because both the soma and the processes were thicker. The arbor area was larger in the SOD1G93A group (**F**) than in the wild type group (**E**), but the difference was only significant in the inferior sector of the OPL. The increase in the arbor area is demonstrated by the larger size of the polygons due to the longer processes. Outer plexiform layer (OPL), and inner layer complex (ILC) (constituted by an inner plexiform layer and a nerve fiber–ganglion cell layer). Number of retinas used in the experiment, WT: *n* = 6; and SOD1G93A: *n* = 6.

In the area occupied by microglial cells, a comparison between the SOD1G93A group and the WT group showed: (i) in the OPL, a significant increase in the total value (*p* < 0.01), as well as in all retinal sectors analyzed (inferior, nasal, and temporal) (*p* < 0.05) (Figure 5D and Figure 6A,B), except in the superior sector; and (ii) in the ILC, a significant increase in the total value (*p* < 0.05) (Figure 5E and Figure 6C,D)).

#### 2.3.3. Arbor Area of Microglial Cells

In the OPL and the ILC, no significant changes were found in the SOD1G93A group in comparison to the WT group, except in the inferior sector in the OPL, in which the arbor area of microglial cells in the SDO1G93A group was significantly larger than that in the WT group (*p* < 0.05) (Figure 5F–G and Figure 6E,F).

### 2.4. Quantitative Study of Brn3a+ RGCs

**Figure 7:**
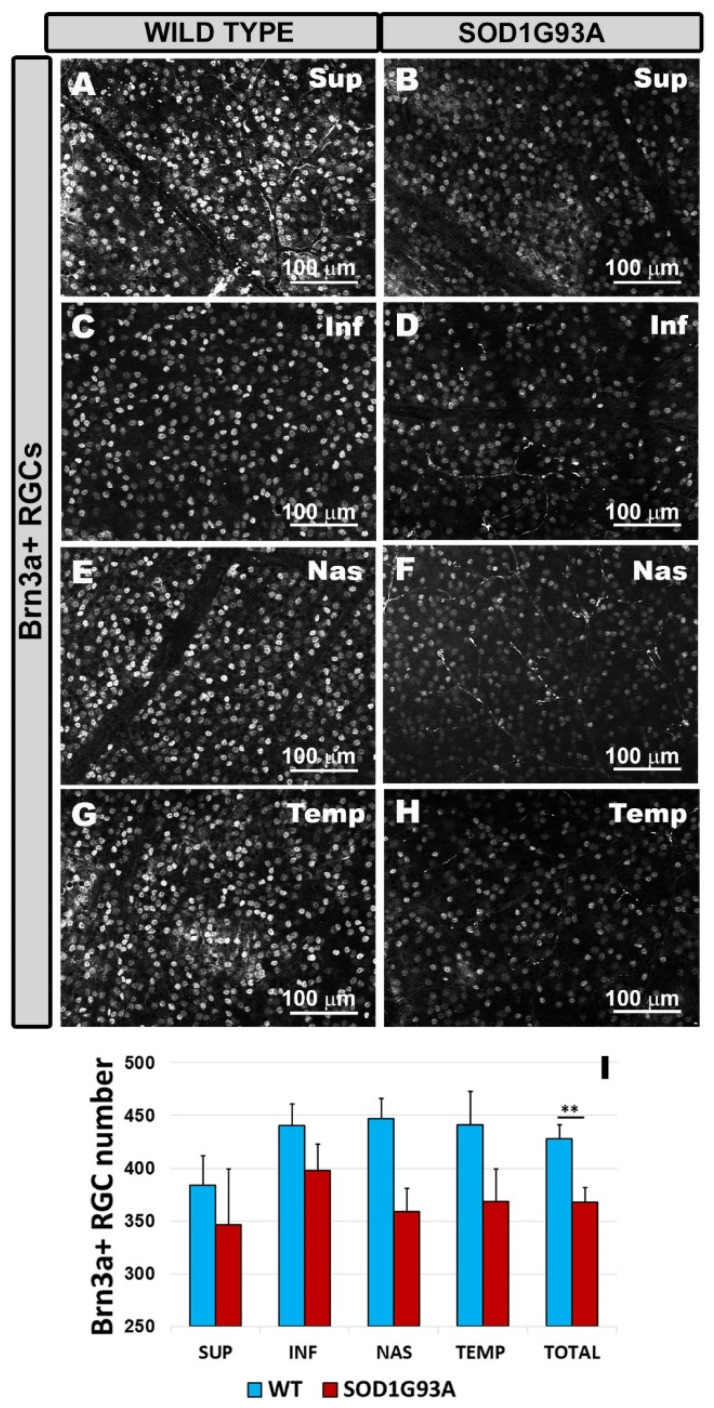
Number of Brn3a+ RGCs in the different retinal sectors superior (**A**,**B**), inferior (**C**,**D**), nasal (**E**,**F**), and temporal (**G**,**H**). Retinal whole-mounts are labeled with anti Brn3a in the wild type group (**A**,**C**,**E**,**G**) and the SOD1G93A group (**B**,**D**,**F**,**H**). The histogram shows the mean number (±standard deviation, SD) of Brn3a+ RGCs per area of 0.1502 mm^2^ in the RGC layer (I). ** *p* < 0.01. Retinal ganglion cell (RGC). Wild type (WT). Superior (SUP), inferior (INF), nasal (NAS), and temporal (TEM). Number of retinas used in the experiment, WT: *n* = 6; and SOD1G93A: *n* = 6. Statistical test: Mann–Whitney U test.

The SOD1G93A group (Figure 7B,D,F,H,I) showed a significant reduction in the number of Brn3a+ RGCs compared to the WT group (Figure 7A,C,E,G,I) when the total value was assessed (*p* < 0.01). The analysis by retinal sectors (superior, inferior, nasal, and temporal) showed a decrease in the number of Brn3a+ RGCs in the SOD1G93A group (Figure 7B,D,F,H,I) with respect to the WT (Figure 7A,C,E,G,I), although the differences did not reach statistical significance.

## 3. Discussion

This study showed for the first time in an SOD1G93A mouse model of ALS at a very advanced stage of the disease (120 days) that there is a loss of RGCs and an activation of microglial cells in the retinal tissue. The signs of microglial activation were found in different retinal sectors (superior, inferior, nasal, and temporal) of the different retinal layers, OPL and ILC. Microglial activation was characterized by (i) a significant increase in the area occupied by each microglial cell, (ii) a significant increase in the microglial arbor area in a sector of OPL, (iii) the presence of cells with shorter processes, and (iv) areas of cell clusters in some retinal areas. In addition, the microglia were labeled with anti-IFN-γ and anti-IL-1β (characteristic of the M1 or pro-inflammatory phenotype), but not with anti-arginase-I and anti-IL-10 (typical of the M2 phenotype).

The SOD1G93A mouse model is one of the most widely used models for the preclinical study of ALS because the animals have similar phenotypes to those of ALS patients [8]. They have a reduced life span of 150 days, so the animals used in this study, which were 120 days old, were in a very advanced stage of the disease.

ALS also affects the visual system. Recently, changes in retinal thickness have been found in ALS patients using OCT [22,26]. There are very few works focused on the study of retinal histological changes in patients and animal models of ALS [22,23,27,28,29]. In the SOD1G93A model (the same model as our study), vacuolization was observed in IPL cells, mainly in the dendrites of the RGCs [27]. In our study, we found a significant decrease in the number of Brn3a+ RGCs (total value) after 120 days of the disease, which coincided with the damage observed in the RGCs in other ALS models [22,27,28], as well as with the thinning of the NFL observed by OCT in patients with ALS compared to the controls [21,22,24,25,26]. These data indicate that in ALS, not only are the motor neurons affected but a loss of RGCs is also produced.

The motor neuron cell bodies are known to be the key sites for the pathogenesis of ALS. However, glial cells in the central nervous system (CNS) are also involved in this condition [30]. Astrocytes and activated microglia have been shown in SOD1 mutant mice to contribute to the progression, but not the onset, of the disease [31,32,33].

In ALS, microglial activation and proliferation has been observed in areas of significant motor neuron loss, such as the motor cortex, the motor nuclei of the brain stem, the corticospinal tract, and the ventral horn of the spinal cord [34,35,36,37,38], but also in areas with mild degeneration [39]. In the SOD1 model, it was found that the overexpression of mutant SOD1 in glial cells contributes to the damage of motor neurons and that the degree of neuronal injury depends on the degree of glial cell pathology [40]. The microglial cells of the SOD1 mutant mice undergo changes in their morphology with gradations from resting microglia to macrophage amoebic forms [35]. In addition, there is also an increase in the number of microglial cells in symptomatic SOD1 transgenic mice, mainly due to the proliferation of the resident microglia [41].

There are only two works that analyzed the behavior of microglial cells in retinal tissue. In the Ranbp2 ALS model, activation of the branched microglia CD11b+ and CD45+, an increase in the number of microglia F4/80+, and development of the amoeboid microglia F4/80+ were observed [28]. However, in the SOD1G93A ALS model (used in this study), Ringer et al. [27] did not observe microglial activation using anti Iba-1 in retinal sections at the onset or at very advanced stages of the disease. They found no increase in the number of microglial cells or any morphological changes in the astrocytes or microglial cells. However, the authors did not rule out the possibility that the microglia were undergoing functional changes (in the cytokines) related to the inflammatory process. However, the neuronal changes observed in this SOD1G93A ALS model in the brain at 50 days of life were followed by microglial morphological changes at 60 days [42,43,44]. Therefore, the authors concluded that if there is an inflammatory process in the retina, the microglia would express a different, less reactive, or even neuroprotective phenotype [27]. In our retinal whole-mounts of SOD1G93A mice, we found changes in the microglial cells of all retinal layers, except for OS. In WT animals, there were very few microglial cells in the OS, and they did not change their appearance or number in the SOD1 mutants. This could indicate that the outermost layers of the retina would not be affected, as observed by Ringer et al. [27], and also that the outer blood–retinal barrier (BRB) would not be compromised in these animals. When the external BRB is altered, as in the model of laser-induced ocular hypertension [45,46,47,48,49], morphological changes and an increase in the number of microglial cells in the OS layer are produced.

Similar to Ringer et al. [27], we found no changes in the number of microglial cells in either OPL or ILC, but we did find other signs of microglial activation in 120-day-old animals (advanced stage of the disease). These changes included an increase in the microglia area; an increase in arbor area, cell displacement, and clustering; and few cells with shorter processes, possibly due to retraction. The reason Ringer et al. [27] found no morphological changes in microglial cells in this same experimental model may be due to the fact that they used retinal sections while we used retinal whole-mounts. Retinal sections do not allow the microglial cell to be observed in its entirety. On the contrary, retinal whole-mounts allow the entire cell to be visualized along the total retinal length, providing very accurate data on the cell’s distribution and morphology, such as the microglia area, the arbor area, cell migrations, and a very accurate count of the number of microglial cells in each of the retinal layers where the microglial cells are located.

It is known that microglia form plexuses in OPL and IPL; both plexuses can be interconnected through changes in the orientation of some microglial cells (from parallel to perpendicular to the retinal surface) [45,46,48]. In our retinas, activated microglia of ILC may have changed their orientation (some of the cells had the shortest processes), thereby interacting with the microglial plexus of OPL and inducing activation of the OPL microglia. This was observed in the microglia of the retinas of a transgenic Alzheimer disease model (3xTg-AD mice) [50] and also in the retinas of mice with laser-induced ocular hypertension (glaucoma model) [45,46,48]. In the latter model, the main damage was produced in the RGCs, and yet microglial activation was observed in all retinal layers (OS, OPL, IPL, and NFL-GCL).

In our study, the area of microglial cells in the OPL and ILC was significantly increased as the total retina was analyzed. When the microglia is activated, it undergoes morphological changes, and is able to release products that induce an inflammatory response [14]. These morphological changes are gradual, moving from a branched resting state to an early activated or “primed” intermediate state until reaching the phagocytic amoeboid state [51]. In the “primed” state, the microglia undergo a thickening of the cell body and the proximal processes, intensifying local surveillance. This transformation can occur in response to primary stimuli derived from neurons or astrocytes, including IFN-γ, tumor necrosis factor α (TNFα), macrophage colony stimulating factor (M-CSF), and granulocyte-macrophage colony stimulating factor (GM-CSF). For brains with ALS, it was proposed that these factors could be released by the damaged neurons [52] (in our case, the RGCs), which would activate the microglia. Therefore, many of the microglial cells of the SOD1G93A mice in our study could have been in this intermediate state of activation, in which the cells thicken their somas and primary processes and involved an increase in the arbor area. The increase in arbor area is only significant in the inferior zone of the OPL. In both the OPL and the ILC, we observed that some cells had more retracted processes or could have changed their orientation. Thus, in the measurements of the arbor area, we found cells with very large arbor areas and others with very small arbor areas, although the latter were much less evident. This could cause the average of the arbor area in the OPL and ILC to be statistically non-significant in many retinal zones and the standard deviation to be high. Microglial cells with retracted processes may be in a more advanced state of activation, approaching an amoeboid phagocytic state in response to secondary stimuli, such as IL-1β, IL-6, and TNF-α [52].

Microglial cells have two distinct phenotypic states that can exert neurotoxic or neuroprotective responses depending on the physiological conditions in which they are found. These phenotypes are characterized as a continuum between two extreme states of activation: the M1 or cytotoxic state, due to the secretion of reactive oxygen species and pro-inflammatory cytokines, and the M2 or anti-inflammatory state, in which the microglia secretes high levels of anti-inflammatory cytokines and neurotrophic factors [53]. In animal models of ALS (SOD1), during the onset of disease, the microglia of lumbar spinal cords express markers related to the neuroprotective M2 phenotype (Ym1 and CD206). However, in the final stages of the disease, it expresses markers related to the neurotoxic M1 phenotype (high levels of NADPH oxidase 2 (NOX2)) [17]. All this suggests that there is a transformation from a neuroprotective phenotype to a cytotoxic phenotype that induces motor neuron damage. In ALS, the conversion from an anti-inflammatory to a pro-inflammatory state of microglial cells can be mediated by the activation of NF-κB factor [54]. In addition, IL-1β has also been shown to act as a signal for the initiation of neuroinflammation in ALS [55].

In the retina, there are no studies that analyze whether the microglia have an M1 or M2 activation phenotype in animal models of ALS. In this study, we used arginase-I and IL-10 to reflect an M2 response and IFN-γ and IL-1β as a marker for the M1 response. Arginase-I is one of the best characterized markers of M2 [56]. In addition, arginase-I competes with iNOS for arginine, which downregulates the production of nitric oxide [57]. IL-10 is a potent anti-inflammatory cytokine that modulates glial activation and exerts neuroprotective actions [58]. IFN-γ is one of the most important inducers of microglial activation [59]. The microglia polarizes the M1 phenotype when stimulated by IFN-γ and can then produce inflammatory mediators such as IL-1β, IL-6, TNF-α, the chemokine ligand C-C 2 (CCL2), reactive oxygen species (ROS), and nitric oxide (NO) [60]. This cytokine plays a key role in maintaining and amplifying the immune response [61]. IL-1 is a cytokine that regulates inflammatory reactions and is involved in the production of numerous cytokines and inflammatory chemokines, as well as neurotoxic substances, such as COX2, inducible NOS, NO, and IL-6, which are implicated in ALS [55]. Our results showed that in 120-day-old SOD1G93A mice, the microglia were labeled with antibodies against inflammatory M1 cytokines (IFN-γ and IL-1β), but were not labeled with anti-inflammatory M2 cytokines (arginase-1 and IL-10). These results suggest that in an advanced stage of the disease, the retinal microglial cells express an M1 activation phenotype or are in a pro-inflammatory state that is neurotoxic to RGCs, as demonstrated by the loss of these neurons. This is consistent with the results found in spinal cords, where the microglia at the end of the disease showed a neurotoxic M1 phenotype [17].

## 4. Materials and Methods

### 4.1. Animals and Ethics

In this study, we used B6.Cg.-Tg(SOD1*G93A)1Gur/J mice, 120 days of age, and age-matched wild-type animals (WT). The animals were kept in the animal house at the Faculty of Medicine of the University Complutense of Madrid (Spain). The animals were kept under conditions of controlled temperature and light (12 h light/dark cycles and light intensity ranging from 9 to 14 lux). The animals had free access to water and were fed a standardized diet. The study was conducted in accordance with the ethical guidelines of Spanish law and the Guidelines for the Humane Endpoints for Animals Used in Biomedical Research. This study was previously approved by the Ethics Committee for Animal Research of Complutense University (Madrid, Spain) and also by the Directorate General of Agriculture and Food, Ministry of Economy, and Employment of the Community of Madrid (approval ID number: 059/16). All animal procedures were performed according to the European Union institutional guidelines for the use of animals in research and in accordance with the Association for Research on the Vision and Ophthalmology (ARVO) standards for the use of animals in research.

### 4.2. Experimental Groups

The animals were divided into two groups: an age-matched control (WT, *n* = 9) and the SOD1G93A group (*n* = 9). Only one of the two eyes was used in the study.

### 4.3. Immunohistochemistry

The animals were sacrificed by decapitation. No perfusions were performed because several tissues of the animals were used for other studies in which fixation could not be used. Prior to removal of the eyeballs, a stitch was placed in the upper eyelid to maintain the orientation of the eyeballs, and the nasal caruncle and insertion of the extraocular rectus muscles were also used to assist this orientation [45]. Once the eyes were extracted, they were incised in the scleral–corneal limb to facilitate the entry of fixative into the eyeball, and then they were fixed by immersion by introducing them into 4% paraformaldehyde in a 0.1 M phosphate buffer, pH 7.4, at 4 °C overnight. The eyes were subsequently washed with phosphate buffered saline (PBS), after which the retinas were separated. The vitreous humor was removed using atraumatic clamps and Westcott scissors, and a deeper cut was made on the upper side of the retina as a mark to maintain each retina’s orientation. The retinas were then prepared to perform the retinal whole-mounts.

To analyze the different morphological signs of microglial activation, we used the antibody against ionized calcium binding adaptor molecule 1 (Iba-1), which allows morphological study of the microglia [62]. To assess the phenotypes of the activated microglial cells, we used antibodies against INFγ and IL-1β as M1 markers and antibodies against arginase-I and IL-10 as M2 markers. To quantify the number of RGCs, we used an antibody against the brain-specific homeobox/POU domain protein 3A (Brn3a), a marker that is located in the nuclei of RGCs that decreases its expression when a cell dies [63] (Table 1).

Retinal whole-mounts were immunostained according to previously used protocols [46,47,48]. In summary, retinas of WT (*n* = 6) and SOD1G93A (*n* = 6) were double-immunostained with rabbit anti-Iba-1 and mouse anti-Brn-3a (Table 1). Retinas of WT (*n* = 3) and SOD1G93A (*n* = 3) were divided into four portions, and each was double-immunostained with rabbit anti-Iba-1, and anti-IFN-γ, anti-IL-1β, anti-arginase-I, or anti-IL-10 (Table 1). The respective secondary antibodies for each primary antibody used in the study are included in Table 1. To dilute the primary antibodies, a solution containing 1% of the serum of the animal where the secondary antibody was developed along with triton-x 100 and PBS was used. The secondary antibodies were diluted in PBS. Three negative controls were used to check that the secondary antibodies reacted with their corresponding primary antibodies. In the first control, the primary antibody was not added. The tissue was incubated with the primary antibody diluent and then with the secondary antibody. In the second control, the secondary antibody was omitted and incubated only with the primary antibody and the secondary antibody diluent. In the third control, neither the primary nor the secondary antibody were used, and the tissues were incubated in the corresponding diluent solutions to evaluate the amount of endogenous fluorescence in the tissue (Figure S1).

The retinal whole-mounts were photographed and analyzed using the Apotome module (Carl Zeiss, Munich, Germany) and a high resolution digital monochrome camera (Cool-SNAP Photometrics, Tucson, AZ, USA), which captured grayscale images; both were coupled to a fluorescence microscope (Axioplan 2 Imaging Microscope Carl Zeiss, Munich, Germany), as described in previous works [46,47,48]. The microscope was equipped with a Zeiss filter set 64 for Alexa Fluor 594 and a Zeiss filter set 10 for Alexa Fluor 488. The apotome module allows one to obtain optical sections of the tissue under conventional fluorescence microscopy via the method of “structured illumination,” which improves the resolution and contrast along the optical axis. The obtained z-stacks were analyzed with Axiovision v. 4.2 (Carl Zeiss, Munich, Germany). To identify the differences in the labeling of the antibodies anti-INFγ, anti-IL-1β, anti-arginase-I, and anti-IL-10, the samples were photographed with equal values of exposure time and excitation intensity for both the WT group and the SOD1G93A group. The figure panels were then assembled using Adobe Photoshop CS3 Extended 10.0 (Adobe Systems, Inc., San Jose, CA, USA).

### 4.4. Quantitative Retina Analysis

#### 4.4.1. Microglial Cell Quantification

In the retinal whole-mounts, we quantified the morphological signs of microglial activation, including the following parameters: (i) the number of microglial cells in the OS, OPL, and the complex of the inner retinal layers (ILC), constituted by the IPL (inner plexiform layer) and the NFL-GCL (nerve fiber layer–ganglion cell layer); (ii) the area occupied by each microglial cell in the OPL and ILC; and (iii) the arbor area of microglial cells in the OPL and ILC. For the count, we combined the IPL and the NFL-GCL because they were very close together, making it difficult to differentiate one layer from the other in the retinal whole-mount. However, the inner nuclear layer separated the OPL from the ILC. This provided us with sufficient micron separation between the two layers, allowing us to see each microglial plexus without any overlap between them in the retinal whole-mount. The microglial cells were arranged parallel to the surface of the retina, which facilitated their complete visibility in retinal whole-mounts. We placed the retinas with the vitreous side up, so that when we started focusing on the whole retinal mount, the first microglial plexus would be the ILC, followed by the OPL plexus and, finally, the few microglia of the OS. This method made it very easy to differentiate the different microglial plexuses in the retinal whole-mounts. In addition, the microglial cells presented different morphologies in each layer of the retina, and this helped us to differentiate the retinal layer that we were studying in the retinal whole-mount.

In each of the retinal whole-mounts of the groups SD1G93A (*n* = 6) and WT (*n* = 6), all the above-mentioned quantifications were carried out. These quantifications were done according to the protocols previously developed by our group [46,64]. In brief, contiguous and equivalent retinal fields on the horizontal and vertical meridians (crossing the optic nerve) were photographed at 20× on each retinal whole-mount using the motorized microscope stage. Each field provided an area of 0.1502 mm^2^. These fields included the superior, inferior, nasal, and temporal sectors of the retina along the x–y axis. We obtained an average of five to six complete fields for each sector, which, multiplied by the three retinal layers analyzed (OS, OPL, and ILC), gave a total of about 72 fields per retina. In this way, a large extension of the retina was analyzed. Since we studied six different retinas per group, a total of about 432 fields per group was evaluated.

(1) Microglial Cell Number

The microglial cells of both the WT group and the SOD1G93A group were manually counted in each of the fields of the different layers of the retina analyzed (OS, OPL, and ILC). Cells located at the edge of the counting field were only counted if their soma was located within the counting area. To perform this counting, we used the interactive tool for manual counting in the AxioVision Release 4.8.2 software (Zeiss, Munich, Germany), “Interactive Measurement,” which was associated with the fluorescence microscope.

(2) Area Occupied by Each Microglial Cell

**Figure 8:**
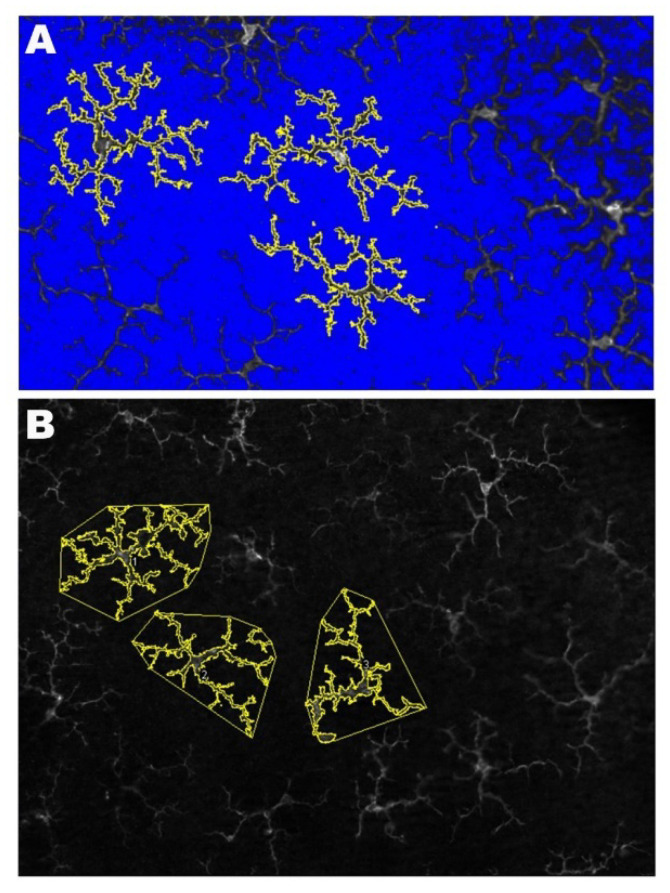
Methodology for the quantification of the microglial cells. (**A**) The photomicrograph shows the results of processing with the Image J program to analyze of the area occupied by each microglial cell. (**B**) The photomicrograph illustrates the method used for the quantification of the arbor area using the Image J program. By using the polygon tool, the most distal tips of the microglial processes were connected.

The area occupied by each microglial cell allows us to analyze if a cell has increased the thickness of its soma and its processes. If the soma and processes are thicker, they will occupy a larger area than if they are thinner (Figure 8A).

To analyze the area of each microglial cell, the Image J program (v. 1.52u 2020) (National Institutes of Healt, Bethesda, MD, USA) was used, which is a Java program domain for image processing. For this purpose, we used each of the five to six fields obtained for each sector (superior, inferior, nasal, and temporal) from both the OPL and the ILC. Three complete randomly selected microglial cells were measured in each of these fields. The only selection criteria used was that the cell had to be complete, that is, that the entire soma and processes had to be within the field of analysis.

First, we turned the images into grayscale to provide the best visibility of all positive staining; then, we adjusted the brightness/contrast to obtain images in which the entire microglial cells and their processes could be correctly visualized. We used the threshold tool to convert the images into binary, thus making the positive staining more evident, and used the “wand tool” to select each microglial cell whose area we wanted to measure. After the microglial cell was selected, the positive stained area was quantified using the “ROI manager,” which allowed us to obtain the total area occupied by the microglial cells (Figure 8A). This process was done for both the WT group and SOD1G93A.

(3) The Arbor Area of Microglial Cells

The arbor area of the microglial cells allows us to analyze changes in process length, regardless of whether the cell is thicker or thinner. If the processes are longer, the arbor area will be larger than if the processes are shorter (Figure 8B).

In the same fields used for “the area occupied by each microglial cell” and using the same processing methods for the images, the “arbor area of the microglial cells” was quantified in three complete microglial cells randomly selected for both OPL and ILC. As for the “area occupied by each microglial cell,” the only selection criterion used was that the cell had to be complete, i.e., the whole soma and processes had to be within the field of analysis. In each cell selected, we used the “polygon tool” to circumscribe the microglial outline with a polygon by connecting the more distal cell processes. The three areas delimited were measured using the “ROI manager,” thus obtaining the total arbor area of each microglial cell (Figure 8B).

(4) Mean IFN-γ and IL-1β Intensity Expression

In the retinal whole-mounts labeled with IFN-γ and IL-1β antibodies, four areas of each retinal whole-mount were randomly selected and were photographed at 20× in the OPL layer. The photographs were taken with equal values of time exposure and intensity of excitation. To quantify IFN-γ and IL-1β expression intensity, we used an algorithm developed in MATLAB (© MathWorks, Inc) and AxioVision 4.8.2 software (Carl Zeiss, Munich, Germany) that is associated with the Apotome device and the fluorescence microscope. The algorithm allows us to identify different intensity levels of IFN-γ and IL-1β expression in the two groups of study. Image Average Intensity was calculated by adding the pixel values of the green channel (IFN-γ or IL-1β immunostaining) of each image and dividing this result by the total number of pixels of the image. The percentages we show in this paper are the result of normalizing the Image Average Intensity values by 255 that is the maximum value each pixel can represent.

#### 4.4.2. Brn3a+ RGC Quantification

We quantified the number of Brn3a+ RGCs in the WT (*n* = 6) and SOD1G93A (*n* = 6) groups following the protocol previously established by our group [47]. In each retinal whole-mount, we selected and photographed (at 20× magnification) equivalent fields of the retina in the RGC layer on both the horizontal and vertical meridians that crossed the optical disc. These included the superior, inferior, nasal, and temporal areas of the retina. We obtained an average of five to six complete fields for each sector, which gave a total of about 24 fields per retina. All fields were contiguous to guarantee that no portion of that meridian of the retina would be lost or duplicated. Each field provided a retinal area of 0.1502 mm^2^. Since we had six retinas per group, a total of about 144 fields per group was evaluated. The RGCs were counted manually in each of the RGC layer fields. For this, we used the interactive tool for manual counting in the AxioVision Release 4.8.2 (Zeiss, Munich, Germany) software “Interactive Measurement,” which was associated with the fluorescence microscope.

### 4.5. Statistical Analysis

Data are shown as the mean ± SD (standard deviation) and were processed by SPSS Statistics 25 (IBM, Chicago, IL, USA), considering statistical signification at *p <* 0.05. The parameters analyzed were the microglial cell number, the area occupied by microglial cells, the arbor area of the microglial cell, and the RGC number. The differences between the WT and SOD1G93A groups were statistically studied using a Mann–Whitney U test.

## 5. Conclusions

We conclude that in the SOD1G93A model of ALS, in advanced stages of the disease (120 days), there are changes in the retinal tissue, such as a loss of RGCs and an activation of microglial cells. The latter affects the OPL and the ILC (IPL, NFL-GCL). The microglia are in M1 activation phenotype or pro-inflammatory state that are neurotoxic to RGCs and could help cause this neuronal loss.

## Figures and Tables

**Table 1:** Antibodies employed for the immunostaining analysis.

Color	Primary Antibody	Conc.	Secondary Antibody	Conc.
GREEN	Rabbit polyclonal anti IL1β (ref. ab9722, Abcam plc, Cambridge, UK)	1:125	Goat anti rabbit Alexa Fluor 488 (ref. ab150077, Abcam plc, Cambridge, UK)	1:150
	Rabbit polyclonal anti IFN-γ (ref. ab9657, Abcam plc, Cambridge, UK)	1:150		
	Mouse monoclonal anti Arginase-I ref. 610708; BD Biosciences, San Jose, CA, USA)	1:25	Goat anti mouse Alexa Fluor 488 (ref. A11001 Invitrogen, Paisley, UK).	1:150
	Rat monoclonal anti IL10 (ref. ab189392, Abcam plc, Cambridge, UK)	1:100	Goat anti rat Alexa Fluor 488 (ref. ab150165, Abcam plc, Cambridge, UK)	1:150
	Mouse monoclonal anti Brn-3a (ref. MAB1585, Sigma-Aldrich, Darmstadt, Germany)	1:300	Goat anti mouse IgG1 Alexa Fluor 594 (ref. A21125, Invitrogen, Carlsbad, CA, USA)	1:150
RED	Rabbit polyclonal anti Iba1 Red Fluorochrome 635 conjugated (ref. 5100756, Wako Chemicals GmbH, Neuss, Germany)	1:100	-	-
	Rabbit polyclonal anti-Iba-1 (ref. 01919741, Fujifilm Wako pure chemical corporation, Osaka, Japan)	1:600	Donkey anti rabbit IgG Alexa Fluor 594 (ref. A21207; Invitrogen, Paisley, UK).	1:800

Commercial antibodies employed, indicating the concentration at which they were used. These include the following: for the determination of cytokines (interleukin 1 beta (IL-1β), interferon gamma (IFN-γ), and interleukin 10 (IL-10)); an M2 marker (arginase- I); and those used to identify microglial cells (Iba-1) and retinal ganglion cells (Brn3a). The color (green/red) indicates how the immunostaining was labeled.

## Data Availability

The data supporting the findings of this study are available from the corresponding author upon request.

## Supplementary Materials

The following are available online at https://www.mdpi.com/1422-0067/22/4/1663/s1, Figure S1: Negative controls of the immunohistochemical labelling.

## Abbreviations

ALS	Amyotrophic Lateral Sclerosis
BRB	Blood-Retinal Barrier
CNS	Central Nervous System
FALS	Familiar Amyotrophic Lateral Sclerosis
G93A	Glycine 93 into Alanine
GCL	Ganglion Cell Layer
GM-CSF	Macrophage Colony Stimulating Factor
Iba-1	Ionized calcium binding adaptor molecule 1
IFN-γ	Interferon γ
ILC	Inner Layer Complex
IPL	Inner Plexiform Layer
IL-1β	Interleukin-1β
M-CSF	Macrophage Colony Stimulating Factor
NFL	Nerve Fiber Layer
NOX2	NADPH oxidase 2
OCT	Optical Coherence Tomography
OPL	Outer Plexiform Layer
OS	Photoreceptor Outer Segment layer
PBS	Phosphate Buffer Saline
Ranbp2	Ran-binding protein2
RGCs	Retinal Ganglion Cells
SALS	Sporadic Amyotrophic Lateral Sclerosis
SOD1	Superoxide Dismutase 1
TNFα	Tumor Necrosis Factor α
UBQLN2	Ubiquilin 2
WT	Wild-Type
