# Supplementary material for: Retinal Ganglion Cell Loss and Microglial Activation in a SOD1G93A Mouse Model of Amyotrophic Lateral Sclerosis
Source: Int J Mol Sci. 2021 Feb 7;22(4):1663. doi: 10.3390/ijms22041663 (PMC7915199; doi:10.3390/ijms22041663)
Supplement: Supplementary file 1 [file ijms-22-01663-s001.pdf]

Supplementary Material

Retinal ganglion cell loss and microglial activation in a SOD1G93A mouse model of amyotrophic lateral sclerosis.

Pilar Rojas [1,4,†], Ana I. Ramírez [1,2,3,†], Manuel Cadena[4], José A. Fernández-Albarral [1], Juan J. Salazar [1,2,3],
Elena Salobrar-García [1,2,3], Inés López-Cuenca [1], Irene Santos-García[5], Eva de Lago[5], José L. Urcelay-Segura[4,6], José M. Ramírez [1,2,6], Rosa de Hoz [1,2,3,*], and Juan J. Salazar [1,2,3,*]

[1] Instituto de Investigaciones Oftalmológicas Ramón Castroviejo. Universidad Complutense de Madrid (UCM), 28040 Madrid, Spain; pilar.rojas.lozano@gmail.com (P.R.); airamirez@med.ucm.es (A.I.R.); joseaf08@ucm.es (J.A.F.-A.); elenasalobrar@med.ucm.es (E.S.-G.); inelopez@ucm.es (I.L.-C.); ramirezs@med.ucm.es (J.M.R.).

[2.] OFTARED-ISCIII, IIORC (UCM), 28011,Madrid Spain.

[3.] Facultad de Óptica y Optometría. Departamento de Inmunología, Oftalmología y ORL. UCM, 28037 Madrid, Spain

[4.] Hospital General Universitario Gregorio Marañón, Instituto Oftálmico de Madrid, 28007 Madrid, Spain; cadenamd@gmail.com (M.C.); joseluis.urcelay@salud.madrid.org (J.L.U.-S.)

[5] Instituto Universitario de Investigación en Neuroquímica, Departamento de Bioquímica y Biología Molecular, Facultad de Medicina, UCM; Centro de Investigación Biomédica en Red de Enfermedades Neurodegenerativas (CIBERNED); Instituto Ramón y Cajal de Investigación Sanitaria (IRYCIS); 28040 Madrid, Spain; isantosg@ucm.es (I.S.-G.); elagofem@med.ucm.es (E.dL.).

[6] Facultad de Medicina. Departamento de Inmunología, Oftalmología y ORL. UCM, 28040 Madrid; Spain.

† These authors (P.R. and A.I.R.) contributed equally to this work.

* Correspondence: rdehoz@med.ucm.es (R.dH.); jjsalazar@med.ucm.es (J.J.S)

Keywords: microglia; retina; SOD1G93A mouse model; ALS; retinal whole-mount; microglial activation; retinal ganglion cells; pro-inflammatory M1 phenotype; anti-inflammatory M2 phenotype

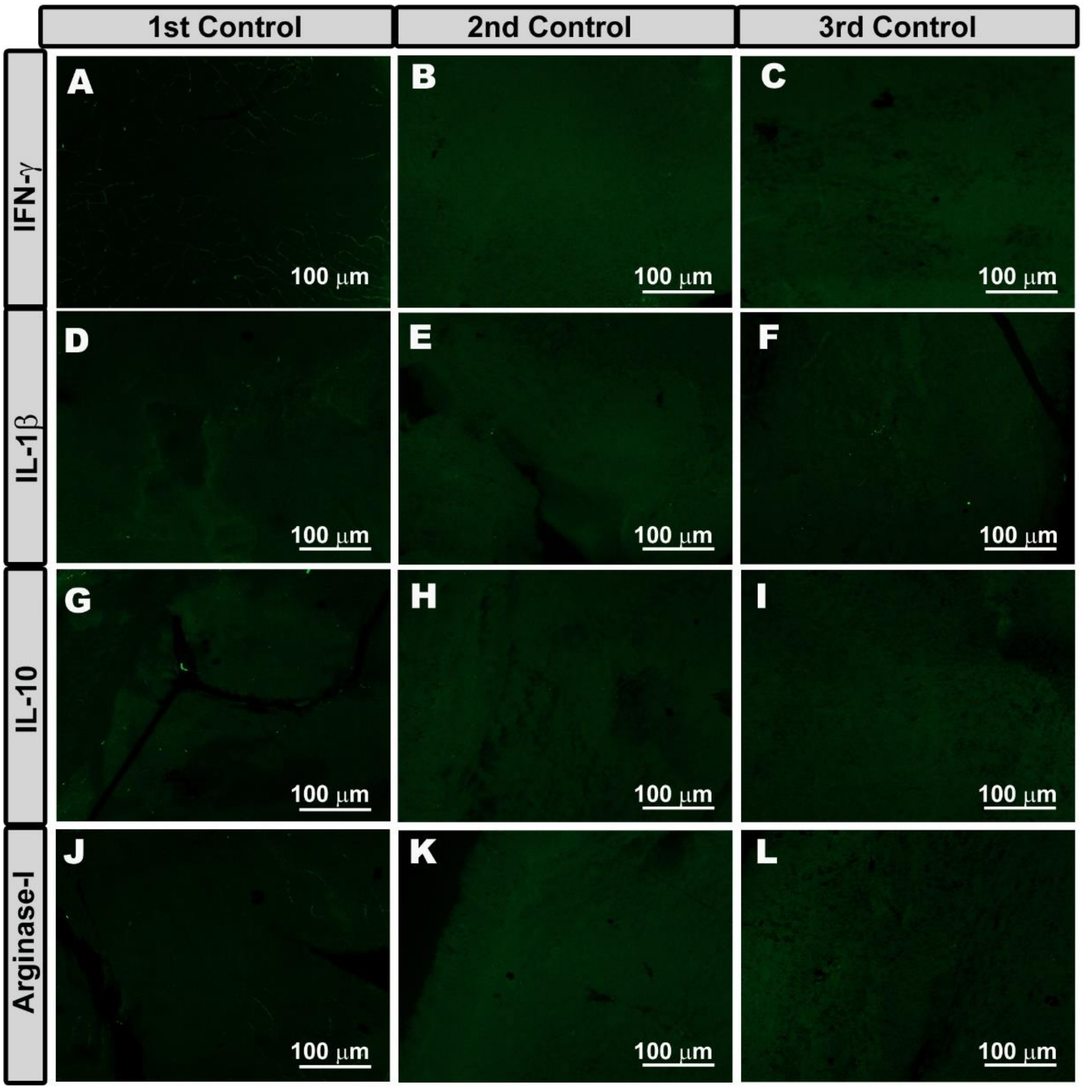

Figure S1. Negative controls of the immunohistochemical labelling of the following antibodies: IFN-γ (A-C), IL-1β (D-F), IL-10 (G-I), arginase-I (J-L). In the 1st control, the primary antibody was not added (A,D,G,J). In the 2nd control, no secondary antibody was added, and only the primary antibody was incorporated into the incubation. In the 3rd control, neither primary nor secondary antibodies were added (C,F,I,L). Some capillaries are visible (arrows) due to the lack of perfusion for tissue fixation (A,J).
